# Scenting Ketones in the Defense Glands of Two Julids From the Caucasus (Arthropoda, Myriapoda, Diplopoda, Julida)

**DOI:** 10.1007/s10886-025-01603-4

**Published:** 2025-04-29

**Authors:** Slobodan E. Makarov, Ljubodrag Vujisić, Günther Raspotnig, Dragan Antić, Felix Anderl, Gordana Krstić, Zvezdana Jovanović, Aleksander Evsyukov, Hans S. Reip, Jelena Milovanović, Bojan Ilić, Vladimir Tomić, Michaela Bodner

**Affiliations:** 1 University of Belgrade – Faculty of Biology, Studentski Trg 16, 11000 Belgrade, Serbia; 2 University of Belgrade – Faculty of Chemistry, Studentski Trg 12-16, 11000 Belgrade, Serbia; 3 University of Graz, Institute of Biology, Universitätsplatz 2/1, 8010 Graz, Austria; 4 University of Vienna – Faculty of Chemistry, Institute of Biological Chemistry & Centre for Microbiology and Environmental Systems Science, Universitätszentrum II (UZA II), Josef-Holaubek-Platz 2, Vienna, Austria; 5 Don State Technical University, Department of Biology and General Pathology, Gagarin Sq.1, Bulding 6, Rostov-on-Don, 344000 Russia; 6 Senckenberg Museum of Natural History Görlitz, Am Museum 1, 02826 Görlitz, Germany

**Keywords:** millipedes, odour, NMR, GC–MS, ketone, Julidone, defensive secretions, Pachyiulini

## Abstract

Millipedes have developed specialized chemical defenses against predators and parasites. Here we investigated the strong repellent odour emitted by two julids from the Caucasus, *Syrioiulus continentalis* (Attems, 1903) and *Pachyiulus krivolutskyi* Golovatch, 1977. By NMR and GC–MS, as well as compound synthesis, we identified the major compound in the secretions of both species as 4-ethylhex-1-en-3-one. A second compound was tentatively identified as the dimer of the main compound as 2-ethyl-1-(6-(pentan-3-yl)-3,4-dihydro-2*H*-pyran-2-yl)butan-1-one. Both compounds amounting for more than 95% of the whole secretion. 4-Ethylhex-1-en-3-one was found for the first time as a naturally occurring compound, and due to its identification in millipedes of the order Julida we propose here the trivial name “julidone”. Considering current knowledge, the replacement of the juliform quinones by novel components such as ketones appears to be highly derived conditions in the chemistry of Julida, possibly triggered by different environmental challenges arising from the epigeic life-style of the two species.

**Supplementary Information:**

The online version contains supplementary material available at 10.1007/s10886-025-01603-4.

## Introduction

Many animals that do not possess mobility, mechanical weaponry, structural defenses, or crypsis utilize chemical means of defense (Berenbaum 1995). These animals defend themselves by compounds that are distasteful, toxic, repellent or otherwise repulsive to consumers and predators. Most defense compounds are secondary metabolites that are not involved in the basic metabolic pathways. Many organisms would probably not be able to survive in their natural environment without the protection provided by secondary chemical compounds.

In the animal kingdom, millipedes (Myriapoda, Diplopoda) are among the most noticeable chemical warriors. The class Diplopoda is the earliest known terrestrial animal group, possessing chemical defenses at least since the Late Ordovician (Edgecombe 2015). This group comprises more than 11,000 species, which are divided into 16 recent orders and 140 families (Enghoff et al. 2015). Eleven orders of millipedes have a chemical defense system in the form of segmentally arranged, paired defense vesicles (ozadenes) localized laterally or dorsally along the body, producing volatile compounds. These exudates usually have a strong odour and protect the specimens from predators and parasites. Some other functions of these compounds, such as the transmission of information, have also been proposed (Makarov 2015). Only a few species release only a single compound from their defensive glands, while in most species it is a mixture of components that varies among taxa. The main chemical compounds involved in the defense of millipedes are quinones, phenols, cyanogenic compounds and alkaloids (Makarov 2015; Shear 2015). However, several compounds from additional chemical classes have been identified in recent years (Bodner et al. 2016, 2017).

**Fig. 1:**
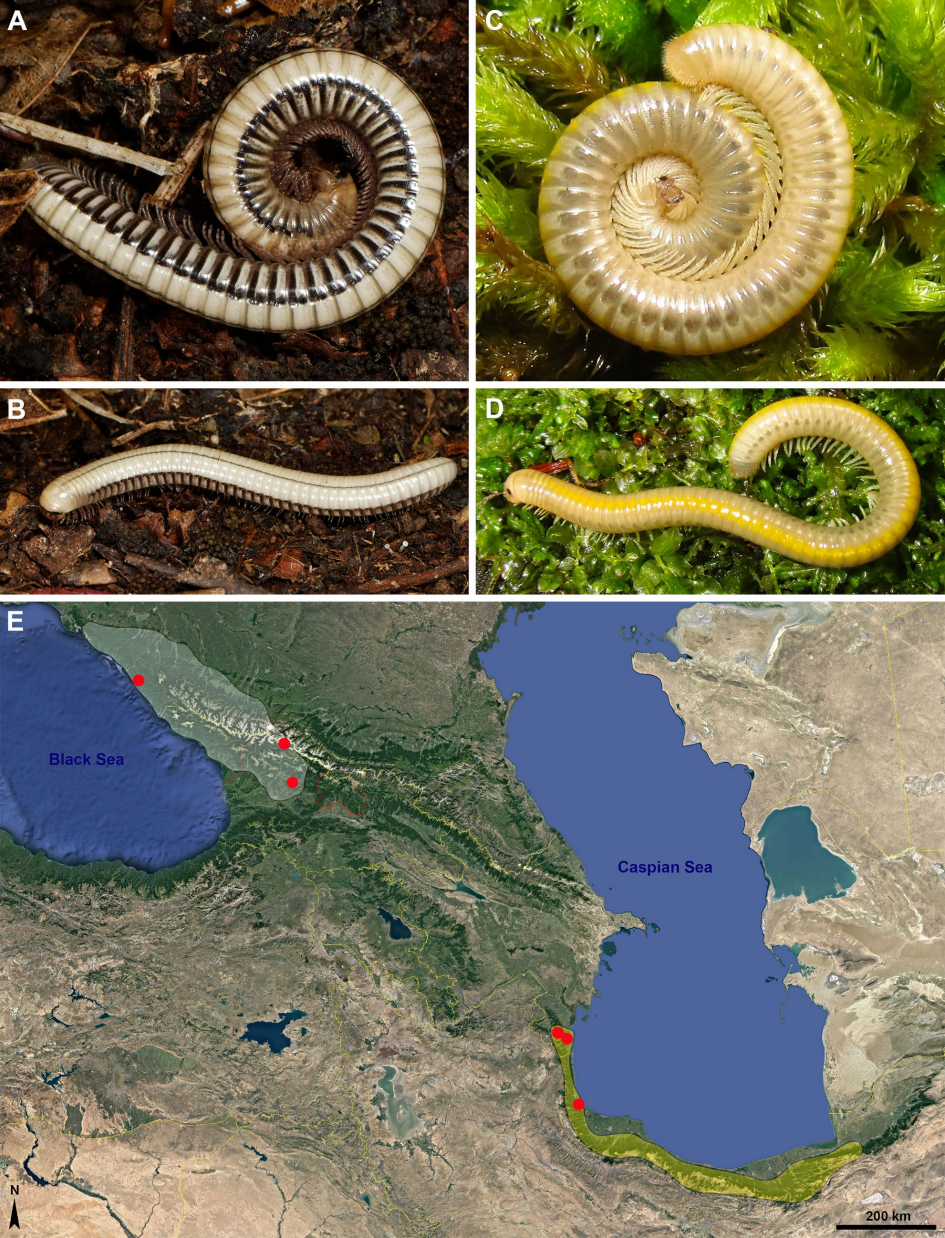
Distribution and habitus of ketone producing millipedes. **A** and **B** *Pachyiulus krivolutskyi* Golovatch, 1977 (photos H. Reip); **C** and **D** *Syrioiulus continentalis* (Attems, 1903) (photos D. Antić); **E** – Distribution: red dots = collection sites (see Material & Methods for explanation)

The release of defensive secretions upon disturbance gives millipedes a characteristic, usually offensive odour. At several localities in the Caucasus, an unusual, flower-like and very strong smell was registered, which, as it turned out, came from millipedes. It bore no resemblance to the odours previously detected in millipedes and could even be noticed from a distance of several tens of meters. We here focused on the identification of the components responsible for this striking odour produced by two species of julids: *Syrioiulus continentalis* (Attems, 1903) and *Pachyiulus krivolutskyi* Golovatch, 1977 (Fig. 1: A-D).

## Methods and Materials

### Collection and Taxonomic Notes on the Material

During field research in Azerbaijan (March 2015) and Iran (April 2017), two of the authors (DA, HR) registered an unusual pelargonium-like strong odour at a few locations. At first, they thought the smell was from a plant. The smell reminded HR of that of *Pachyiulus krivolutskyi* Golovatch, 1977 (Fig. 1: A, B). Following the collection of terrestrial arthropods, the source was identified as the julid millipede *Syrioiulus continentalis* (Attems, 1903) (Fig. 1: C, D) and DR collected samples for chemical analysis of the defensive secretion (Table 1).

**Table 1:** Collection data of analysed specimens

Species	Locality	Specimens
*Syrioiulus continentalis* (Attems, 1903)	1) Azerbaijan, Lerik rayon, Hyrcan Nature Reserve, road Lǝnkǝran-Lerik at km 32, small side valley, forest of *Parrotia persica* with some *Quercus*, thick leave layer, 38.7638, 48.5819, 400 m asl.; 26.03.2015, leg. Antić D. & Reip H	8*
	2) Azerbaijan, Lǝnkǝran rayon, Hyrcan Nature Reserve, Daştatük 1.3 rkmXanbulan Reservoir, *Parrotia persica* forest, divers bushes, in the leaf litter, 38.6747, 48.7622, 110 m asl.; 27.03.2015, leg. Antić D. & Reip H	7*
	3) Iran, Gīlān, Talesh, Gīsūm forest N of SīāhBīl, mossy lowland forest (*Acer*, *Parrotia persica*, *Carpinus*), 37.6554, 48.9853, 40 masl.; 16.04.2017, leg. Antić D. & Reip H	8**
*Pachyiulus krivolutskyi* Golovatch, 1977	4) Russia, Krasnodar Province, Lazorevskoe District, near Tatiànovka, under bark, 450 m asl.; 05.08.2018, leg. Evsyukov A. & Marchenko V	2♂♂
	5) Georgia, Samegrelo-Zemo Svaneti, Mestia, south-east slope below Chalaadi Glacier, various deciduous shrubs with *Abies nordmanni* and *Picea orientalis* and others, in the leaf litter, 43.1147, 42.7469; 09.06.2019, leg. Reip H	1♂
	6) Georgia, Samegrelo-Zemo Svaneti, Nikortsminda, near Nikortsminda Sakinule Cave, deciduous forest, in the foliage/scree mixture, 42.4633, 43.0667; 14.06.2019, leg. Reip H	1♂

^*^ Individuals (including male, females and juveniles) were pooled for extraction, ** males and females were pooled and extracted separately

For *P. krivolutskyi*, Evsyukov (2016) already reported a particularly strong repellent smell. Both species belong to the julid tribe Pachyiulini. *P. krivolutskyi* was originally described as *Iulus foetidissimus* Muralewicz, 1907, which turned out to be a homonym of *Iulus foetidissimus* Savi, 1819. Subsequently, this taxon was listed in the literature under several other names until it was validated by Mauries et al*.* (1997) under the name used today (for details see Evsyukov 2016). Similarly, *S. continentalis* was originally placed in the genus *Pachyiulus* Berlese, 1883 and the subgenus *Dolichoiulus* Verhoeff, 1900 and then in the genus *Amblyiulus* Silvestri, 1896 to finally end up in the genus *Syrioiulus* Verhoeff, 1914 (for details see Vagalinski 2020). Both species are endemic to the Caucasian Ecoregion (Fig. 1: E). *Pachyiulus krivolutskyi* is distributed in the north-western Caucasus, the Republic of Adygea, Krasnodar Province and Karachaevo-Cherkessia (all in Russia), as well as in Georgia (Evsyukov 2016). On the other side, *S. continentalis* can be found in the south-east, on the southern coast of the Caspian Sea, from the Hyrcanian part of Azerbaijan via the Elburz Mountain in Iran probably as far as the border with Turkmenistan (Evsyukov et al. 2021). The length of adult *S. continetalis* is about 40 mm, the width 2.4–2.7 mm. Apart from the black ommatidia and antennae, the body is whitish to pale yellow with a strong and broad dorsal yellow stripe. *P. krivolutskyi* has a length of almost 90 mm and width of 3.4–5.9 mm. Evsyukov (2016) distinguished three colour morphs in *P. krivolutskyi*. The first morph inhabits the northern and central parts of the distribution area. The antennae, most of the head, the anterior leg-pairs, the thin axial line, the longitudinal stripe at the ozopores level on each side and the transverse bands below the ozopores are all black to blackish (Fig. 1 A and B). The second morph has antennae, legs, most of the head, a discontinuous lateral stripe at the level of the ozopores on each side, a thin axial line and transverse bands below the ozopores, all of which are gray or greyish. It appears to be a transitional form between the other two morphs and to inhabit a transitional distribution area (Evsyukov 2016). The third morph inhabits the southern part of the range with antennae, most of the head and a thin axial line in white to yellow colour.

### GC–MS Analysis

Individuals from different localities were whole body extracted either individually or pooled in around 2 mL of dichloromethane (DCM) for 5 min. To eliminate the effects of composition-altering oxidation and degradation of compounds, aliquots of the extracts were immediately analyzed by gas chromatography-mass spectrometry (GC–MS). The GC–MS analyses were performed on two different GC–MS systems: extracts of the pooled individuals of locality 1, 2 and 3 (Table 1) were measured on an Agilent 7890 A GC system equipped with a 5975 C inert XL EI/CI MSD and a FID detector connected by capillary flow technology 2-way splitter with make-up gas. An HP-5MSI capillary column (Agilent Technologies, 0.25 mm i.d., 30 m length, 0.25 μm film thickness) was used. Samples were injected in split-less mode and the injection volume was 1 μL. The carrier gas (He) flow rate was 1.6 mL/min at 40 °C (constant pressure mode). The column temperature was programmed linearly in a range of 40–300 °C at a rate of 10 °C/min with an initial 1-min and a final 8-min hold. Mass spectra were acquired in electron ionization mode (EI) with ion energy of 70 eV and chemical ionization (CI) mode with ion energy of 150 eV. CI mass spectra were obtained in positive mode with isobutane as the reagent gas. The scan range was *m/z* 40–550 in EI mode, and *m/z* 60–550 in CI mode. A library search and mass spectral interpretation was performed using NIST AMDIS (Automated Mass Spectral Deconvolution and Identification System) software, ver. 2.70. The search was performed against our own library containing more than 5,000 mass spectra (more than 100 mass spectra of defensive compounds from arthropods), and the commercially available NIST17, Wiley 07 libraries.

Aliquots (1.5 µL) of *P. krovolutskyi* from sites 5 and 6 (individual whole body extract) were measured on a Trace GC-DSQ I GC–MS system from Thermo Fisher (Vienna, Austria). The GC was equipped with a ZB-5 fused silica capillary column (30 m × 0.25 mm id., 0.25 µm film thickness, Phenomenex, Aschaffenburg, Germany). Injection was splitless with helium as carrier gas at 1.2 ml min^-1^. The temperature of the GC oven was held at 50°C for 1 min and then programmed to 300 °C at 10 °C min^-1^, then held for 5 min at 300 °C. The ion source of the MS and the transfer line were kept at 200 °C and 310 °C, respectively. Electron impact (EI) spectra were recorded at 70 eV. Gas chromatographic retention indices (RI) of compounds were calculated according to Van den Dool and Kratz (1963), using a standard mixture of n-alkanes (C_9_-C_36_) (SigmaAldrich, Vienna, Austria).

### NMR Analysis

In addition to the GC–MS approach, one specimen of *S. continentalis* was soaked in approximately 1 mL of deuterochloroform (CDCl_3_) (Merck, Darmstadt, Germany) for 5 min, then the solution was transferred to a NMR tube by filtration through laboratory tissues set on the tips of a Pasteur pipette. The total volume of filtered sample was 500 µL. A Bruker Avance III (500 MHz) NMR spectrometer (Darmstadt, Germany) with 5 mm BBO probehead was used for ^1^H and ^13^C NMR as well as for 2D NMR experiments: COSY (Correlated Spectroscopy), HSQC (Heteronuclear Single Quantum Coherence) and HMBC (Heteronuclear Multiple Bond Correlation). NMR spectra were recorded by TOPSPIN software (ver. 3.5) and were processed with MestreNova software (ver. 12.0).

### Chemical Synthesis of 4-ethylhex-1-en-3-one

Dry THF wash purchased from Acros/Fisher Scientific in SureSeal® bottles stored over activated 4 Å molecular sieves. All operations involving air and moisture sensitive reagents/intermediates were performed under an atmosphere of dry nitrogen. 3-Bromopentane (7.5 mL, 60 mmol) was slowly added to a suspension of magnesium turnings (1.56 g, 64 mmol) in THF (10 mL). After the reaction started, further THF (40 mL) was added and the reaction mixture was stirred at ~ ambient temperature (cooled by a water bath). In parallel, zinc chloride (8.4 g, 62 mmol) was flame dried twice in vacuo. Thereafter, it was dissolved/suspended in THF (20 mL). After 1 h, the above Grignard solution was added via canula to the zinc chloride suspension/solution and the “Grignard” flask and canula were rinsed with THF (10 mL) and Et_2_O (20 mL). The resulting white suspension was cooled to 0 °C and Pd(PPh_3_)_4_ (80 mg, 0.07 mmol, ~ 0.2 mol%) and acryloyl chloride (2.5 mL, 31 mmol) were added. The reaction mixture was further stirred at 0 °C. After 1.5 h, the reaction mixture was diluted with n-hexane (70 mL) and the resulting mixture was washed with H_2_O (50 mL) and saturated aqueous ammonium chloride solution (50 mL). The organic layer was dried over sodium sulfate and concentrated under reduced pressure to yield a yellow/orange liquid (3.3 g). Purification of the residue by flash chromatography (*n*-hexane:*tert*-butyl methyl ether 29:1 → 14:1) yielded the product as colorless oil (264 mg, 7%). The desired product was the first-eluting component. Its physical properties were in good agreement with the reported values (Ogasawara et al. 2005).

## Results

**Fig. 2:**
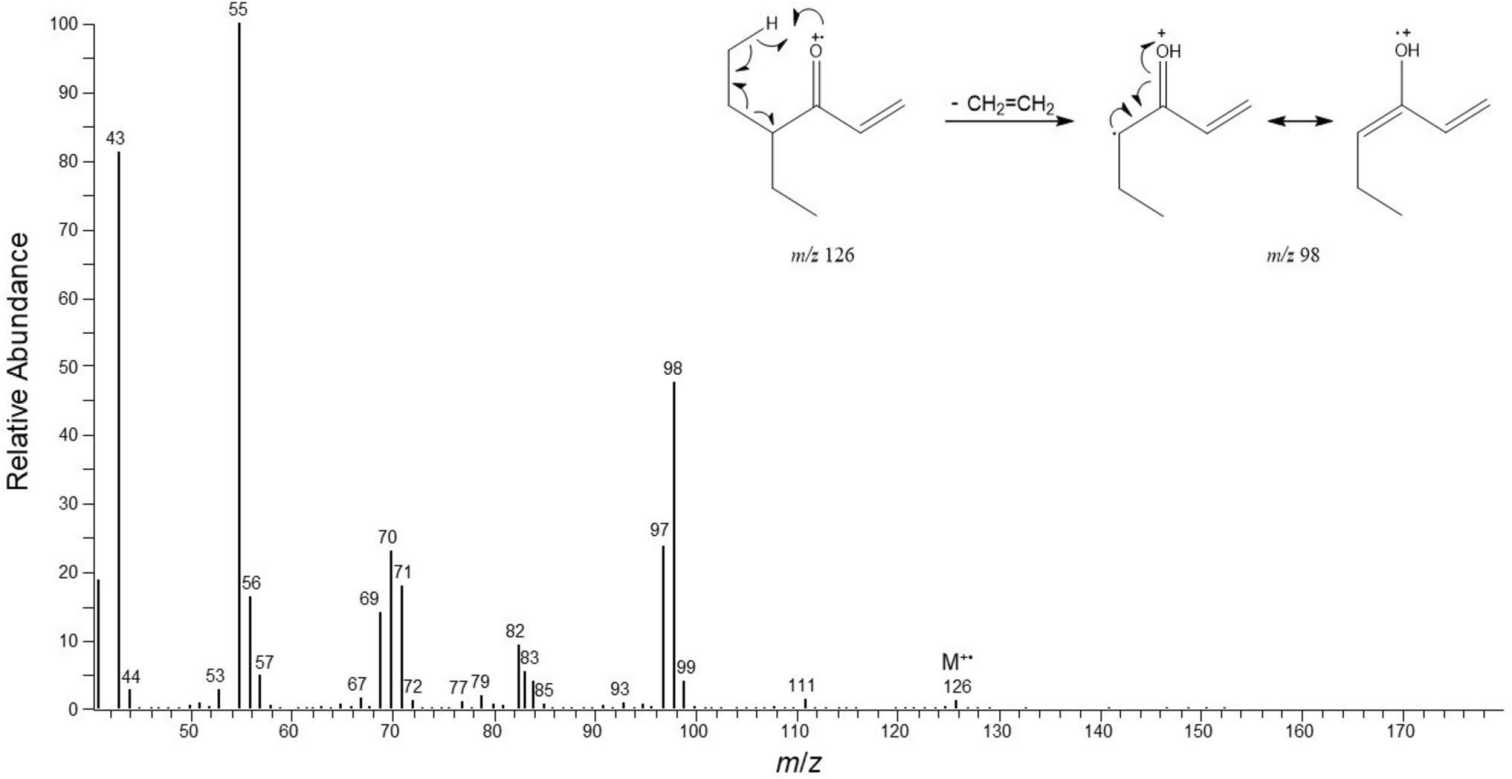
EI mass spectrum of 4-ethylhex-1-en-3-one (compound 1) with explanation for *m/z* 98, the most characteristic fragmentation from McLafferty rearrangement

A total of 4 extracts were analyzed from *S. continentalis* and 3 extracts from *P. krovolutskyi* (Table 1). The chemical profiles of both species were found to be similar, regardless of population and location. By GC–MS, the DCM whole body extracts exhibited two prominent compounds, together amounting for more than 95% of the peak area of the total chromatogram (SI Figure S1, S2). The two compounds showed a characteristic proportion of about 98:2 (Table 2). The main compound was identified as 4-ethylhex-1-en-3-one (**1**). To our knowledge compound **1** is only known as synthetic product and it is the first finding of this vinyl ketone in animals. According to its identification in millipedes of the order Julida, we propose a trivial name of this compound, julidone. Compound **1** was identified by the interpretation of its MS spectrum and NMR data, and its identity was confirmed by compound synthesis. In EI-MS spectrum, α-cleavage results in an abundant fragment at *m/z* 55 (C_3_H_3_O^+^), and McLafferty rearrangement creates an ion at *m/z* 98 (M-28) by losing ethylene (Fig. 2).

**Table 2:** Gas chromatographic^1^ and mass spectral data from analysed species

Peak	RI^2^	mass spectrometric fragmentation *m/z* (rel. intensity)	Compound	*P. krivolutskyi*	*S. continentalis*
1	915	126 (M^+^, 2), 111 (2), 99 (6), 98 (54), 97 (31), 84 (6), 83 (16), 71 (24), 70 (30), 69 (19), 57 (6), 56 (21), 55 (100), 43 (82)	4-ethylhex-1-en-3-one*	97.8	96.8
2	1606	252 (M^+^, 16), 154 (10), 153 (100), 135 (12), 125 (5), 109 (16), 107 (12), 93 (14), 83 (21), 81 (14), 71 (49), 69 (31), 57 (23), 55 (28), 43 (36)	2-ethyl-1-(6-(pentan-3-yl)-3,4-dihydro-2*H*-pyran-2-yl) butan-1-one**	2.2	3.2

^1^Main compounds are given as relative ratios to each other and are representing mean values of all examined extracts: Three extracts of *P. krivolutskyi* (including pooled extract as well as individual extract), and four pooled extracts of *S. continentalis*. ^2^ RI was calculated according to van den Van den Dool and Kratz (1963),^*^RI and mass spectrum showed full correspondence with synthesised compound (SI Figure S3), ^**^tentative

**Fig. 3:**
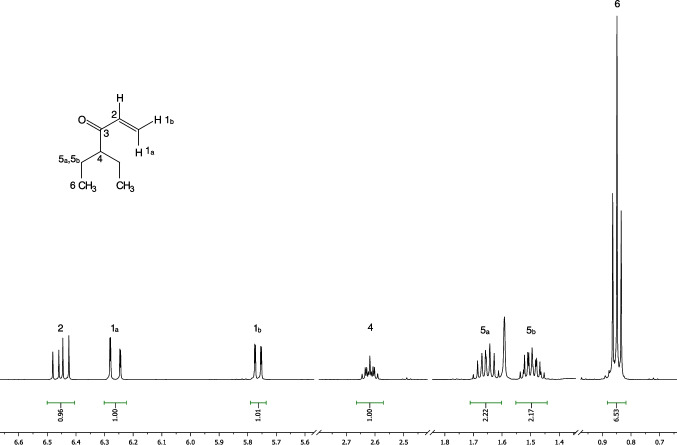
^1^H NMR spectrum (500 MHz, CDCl_3_) combined by four expansions with assignation of all signals of 4-ethylhex-1-en-3-one in the extract of *S. continentalis* (full ^1^H NMR spectrum is presented in SI Figure S5)

In addition, 1D and 2D NMR analyses supported the chemical structure of compound **1** as 4-ethylhex-1-en-3-one (^1^H NMR (500 MHz, chloroform-*d*) δ 0.85 (t, *J* = 7.5 Hz, 6H), 1.49 (dqd, *J* = 14.9, 7.5, 5.5 Hz, 2H), 1.66 (dqd, *J* = 14.9, 7.5, 5.5 Hz, 2H), 2.62 (tt, *J* = 8.1, 5.5 Hz, 1H), 5.76 (dd, *J* = 10.5, 1.3 Hz, 1H), 6.26 (dd, *J* = 17.5, 1.3 Hz, 1H) and δ 6.45 (dd, *J* = 17.5, 10.5 Hz, 1H), ^13^C NMR (125 MHz, chloroform-*d*): δ 11.8, 24.3, 52.3, 127.9, 136.0, and 204.3) (Fig. 3 and Figure S5 – S9). Obtained NMR data are in perfect agreement with those published by Ogasawara et al. (2005). In our case, there is one major compound and all signals are clearly visible in both proton and carbon NMR spectra (Fig. 3, SI Figure S5 and S6), thus providing optimal prerequisites for NMR Basic 2D NMR experiments such as COSY, HSQC and HMBC (Supplementary Figure S7 – S9).

The second compound has only been tentatively identified on the basis of GC-EI-MS data as 2-ethyl-1-(6-(pentan-3-yl)−3,4-dihydro-2*H*-pyran-2-yl)butan-1-one (**2**) (SI Figure S4), which was clearly a minor compound in both *P. krivolutskyi* and *S. continentalis*. We suppose that compound **2** is the hetero-Diels–Alder dimer (HDA) of compound **1**. Several HDA dimers have been registered in harvestmen (Rocha et al. 2013), but at the moment we cannot confirm the possibly enzymatic formation of this compound from compound **1**. EI-MS data for both compounds, julidone and 2-ethyl-1-(6-(pentan-3-yl)−3,4-dihydro-2*H*-pyran-2-yl)butan-1-one, are summarized in Table 2. In addition, [M + H]^+^ ions was supported by positive CI-MS spectra.

## Discussion

### Ketones in the Defensive Secretions of Millipedes

We identified julidone in two pachyiulinine julid species, *Syrioiulus continentalis* and *Pachyiulus krivolutskyi*, as a novel naturally occurring compound. The presence of ketones in defense repertoire of animals is not unusual. In one of the first reviews on defense mechanisms in arthropods, Blum (1981) noted that different arthropods produce more than 50 ketones in the range of C_4_-C_22_-atoms, and that almost 60% of these are idiosyncratic at the familiar level. Subsequently, a large number of new ketones in the C_3_-C_46_ range have been described from various animal taxa (both invertebrates and vertebrates). Up to now, almost 600 different ketones have been recorded in the literature, often acting as pheromones, but also as allomones, attractants or even kairomones (El-Sayed 2024).

Some recent reports have also reported the presence of ketones in the defense secretions of millipedes. Makarov et al. (2010) reported benzyl methyl ketone in *Polydesmus complanatus* (Linnaeus, 1761) and benzyl ethyl ketone in *P. complanatus*, *Brachydesmus dadayi* Verhoeff, 1895 and *B. avalae* Ćurčić & Makarov, 1997. In hexane extracts of the polydesmid millipede *Nedyopus tambanus mangaesinus* (Attems, 1909), Kuwahara and co-workers (2017) identified a cyclic ketone, 1-phenyl-2-pentanone. This ketone was present in all postembryonic stages and showed a sexually biased increase in females after hibernation, authors speculated on a role in sexual communication (Kuwahara et al. 2017). Jones et al. (2018) identified both acyclic, unsaturated ketones in the methanol extracts of six polydesmid *Gasterogramma* species from Tasmania. The pungent odour of these millipedes is due to unsaturated ketones (e.g., vinyl ketones), and the authors speculated that the noxious volatile and chemically reactive nature of these ketones implied a defensive role. In the species herein studied, julidone is the dominant component of the defense exudates with probably protective function in a wide sense, especially considering that vinyl ketones are known as very effective irritants and repellents (Jones et al. 2018). Within the order Julida, ketones were previously only known as minor compounds of two julids from Serbia: 2-heptadecanone from *Megaphyllum bosniense* (Verhoeff, 1897), and 3-octanone and 1-octen-3-one from *M. unilineatum* (C. L. Koch, 1838) (Ilić et al. 2018). Thus, ketones have hitherto only been found in Juliformia and Merocheta; both lineages are the most derived among millipedes.

### Modifications in the Traditional Quinone Repertoire of Julida

The dominance of quinones in the secretions of julids has long been considered a consistent reality in julid defense chemistry (Makarov 2015). However, in the last years, additional components such as alcohols, esters, phenols and aldehydes have increasingly been recognized in the defense exudates of julids. More recent studies even showed chemical profiles without quinones, based on completely aberrant components (Shimizu et al. 2012; Bodner et al. 2016, 2017; Ilić et al. 2018). Regarding the differing chemistry and scattered taxonomic occurrence of ketones in millipedes, ketones appear to have a polyphyletic origin. Some of these may arise from benzoquinone precursors which share similarities to both quinones and ketones (Ilić et al. 2018). The only study on the biosynthesis of vinyl ketones, though not on millipedes, is a recent work by Rocha et al. (2013) who demonstrated that vinyl ketones in harvestmen are of polyketid origin and that both benzoquinones and vinyl ketones share similar precursors. Rocha et al. (2013) proposed the production of vinyl ketones as derived from the ancient state of benzoquinone production. Early studies on benzoquinone origins in millipedes assumed aromatic amino acid precursors (Duffey and Blum 1977; Blum 1981). In any case, it appears that the absence of quinones and the presence of ketones in the two studied julids from the Caucasus are highly derived conditions. In an ecological context, there is some evidence that species that produce benzoquinones live mainly in the soil and seek shelter under stones and tree stumps, as it is the case for most julid species. Conversely, non-quinonic components have frequently been found in species that live above ground or in higher vegetation zones which is a rather rare condition in julids, but it may drive secretion diversification. In the julid *Typhloiulus orpheus* Vagalinski, Stoev & Enghoff, 2015, for instance, a soil surface species, quinonic defense is reduced and replaced by anthranilates, N-containing compounds unique in millipedes (Bodner et al. 2017). Comparably, *Allajulus dicentrus* (Latzel, 1884) produces aliphatic aldehydes but shows a reduced quinone fraction, unlike its congenerics (Bodner and Raspotnig 2012). We currently do not exactly know which ecological conditions induce changes in a conserved and rather stable quinone-producing gland system as in julids, but secretion diversity is likely to be driven by differences in predation pressure in different environments. *Pachyiulus krivoluskyi* is one of the largest millipedes in the area where it was collected and it can be categorized as a stratobiont (Golovatch 1987). Due to its size, it is easily spotted by predators, potentially rendering it an easy prey. *Syroiulus continentalis* is also a relatively large julid that is easily spotted by potential predators due to its colour. Obviously, both species show an aposematic coloration. Red and yellow are known to be effective warning signals, and high contrast colour patterns (black and white) have been shown to be learned more quickly (Zylinski and Osorio 2013; Stevens and Ruxton 2012). *P. krivolutskyi* with black, grey or even yellow stripes and *S. continentalis* with a strong yellow dorsal stripe fit this statement perfectly. Summers and Clough (2001) found that colouration evolved"in tandem with toxicity", leading to a positive correlation between the degree of toxicity and the visual conspicuousness of the animals. With a combination of warning signals and secondary chemical defense, both aposematic julids are possibly able to deter predators multimodally, i.e., by activating their visual and olfactory receptors. Diplopods are preyed by numerous invertebrates as well as by visually predatory vertebrates such as amphibians, reptiles, birds and mammals (Shear 2015). Based on these facts, it may be assumed that high predation pressure resulted in a change in metabolic pathways finally leading to the synthesis of julidone in *P. krivoluskyi* and *S. continentalis*. Julidone has an exceptionally strong odour for humans and can be detected even by the human nose from several tens of meters. The combination of warning colour and secondary chemical defence by julidone could be an early warning system for potential predators, an aposematic scent, as Blum (1981) suggested for some cyanogenic millipedes. However, its exact biological function is still under investigation.

## Supplementary Information

Below is the link to the electronic supplementary material.

Supplementary file1 (PDF 494 KB)

## Data Availability

No datasets were generated or analysed during the current study.
