# Supplementary material for: Scenting Ketones in the Defense Glands of Two Julids From the Caucasus (Arthropoda, Myriapoda, Diplopoda, Julida)
Source: J Chem Ecol. 2025 Apr 29;51(3):50. doi: 10.1007/s10886-025-01603-4 (PMC12040981; doi:10.1007/s10886-025-01603-4)
Supplement: Supplementary file 1 — Supplementary file1 (PDF 494 KB) [file 10886_2025_1603_MOESM1_ESM.pdf]

Supporting Information

Scenting ketones in the defense glands of two julids from Caucasus (Arthropoda, Myriapoda, Diplopoda, Julida)

Slobodan E. Makarov, Ljubodrag Vujisić, Günther Raspotnig, Dragan Antić, Felix Anderl, Gordana Krstić, Zvezdana Jovanović, Aleksander Evsyukov, Hans S. Reip, Jelena Milovanović, Bojan Ilić, Vladimir Tomić, Michaela Bodner

Content:

1) **Supporting Figures – GC-MS**

Figure S1. Total ion GC-EI-MS chromatogram of dichloromethane extract of defensive secretion of *Syrioiulus continentalis*

Figure S2. Total ion GC-EI-MS chromatogram of dichloromethane extract of defensive secretion of *Pachyiulus krivolutskyi*

Figure S3. EI mass spectrum of synthesised 4-ethylhex-1-en-3-one

Figure S4. EI mass spectrum compound 2 in the extraxct of *P. krivolutskyi*

2) **Supporting Figures – NMR**

Figure S5. ^{1}H NMR (500 MHz, $CDCl_3$) spectrum of the *S. continentalis* extract

Figure S6. ^{3}C NMR (125 MHz, $CDCl_3$) spectrum of the *S. continentalis* extract

Figure S7. ^{1}H,^{1}H-COSY (500 MHz, $CDCl_3$) spectrum of the *S. continentalis* extract

Figure S8. ^{1}H,^{13}C-HMBC (500 MHz, $CDCl_3$) spectrum of the *S. continentalis* extract

Figure S9. ^{1}H,^{13}C-HSQC (500 MHz, CDCl3) spectrum of the *S. continentalis* extract

1) Supporting Figures – GC-MS

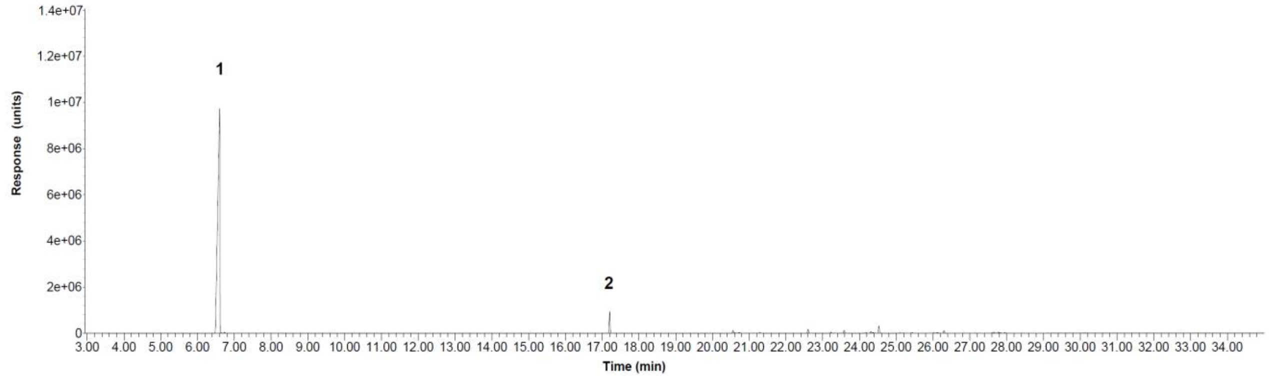

Figure S1. Total ion GC-EI-MS chromatogram of dichloromethane extract of defensive secretion of *Syrioiulus continentalis*

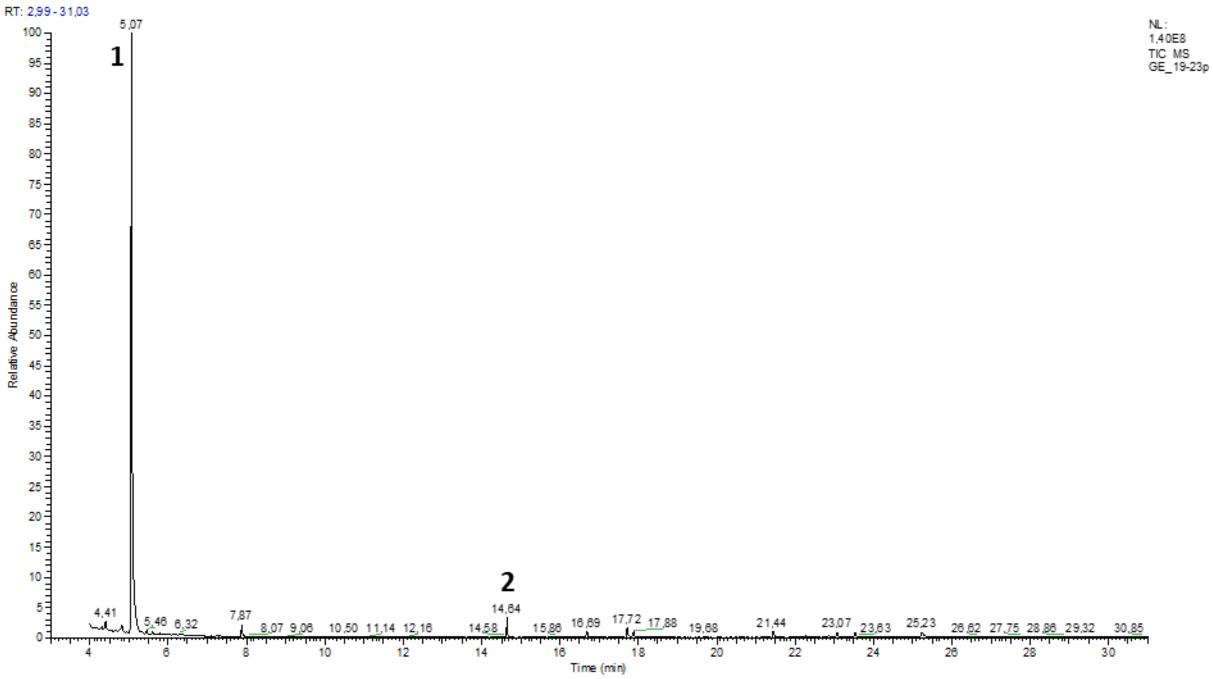

Figure S2. Total ion GC-EI-MS chromatogram of dichloromethane extract of defensive secretion of *Pachyiulus krivolutskyi*

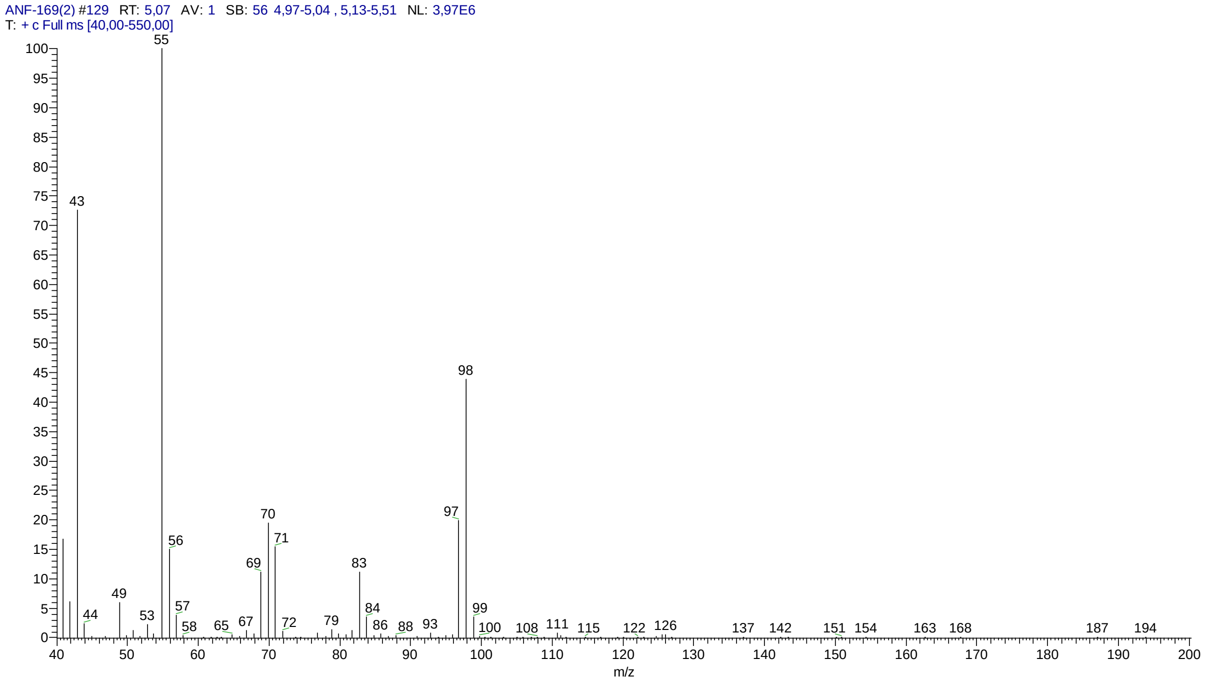

Figure S3. EI mass spectrum of synthesized 4-ethylhex-1-en-3-one (**1**)

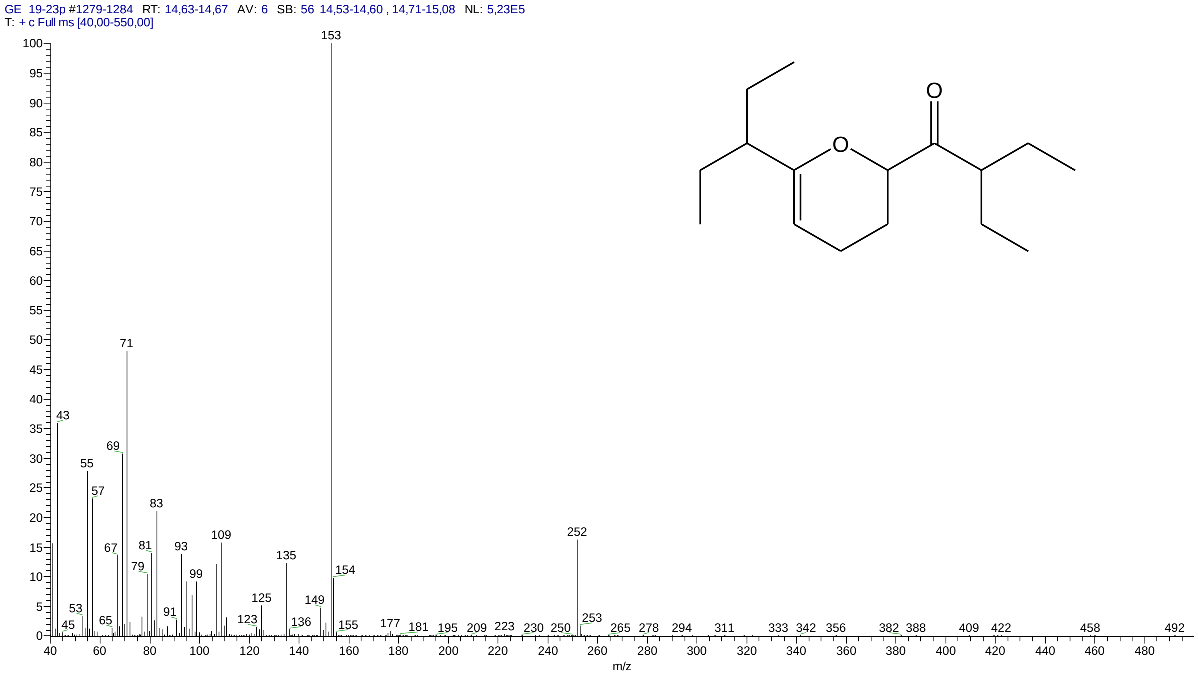

Figure S4. EI mass spectrum of compound **2** in the extraxct of *P. krivolutskyi* with the tentative structure

2) Supporting Figures – NMR

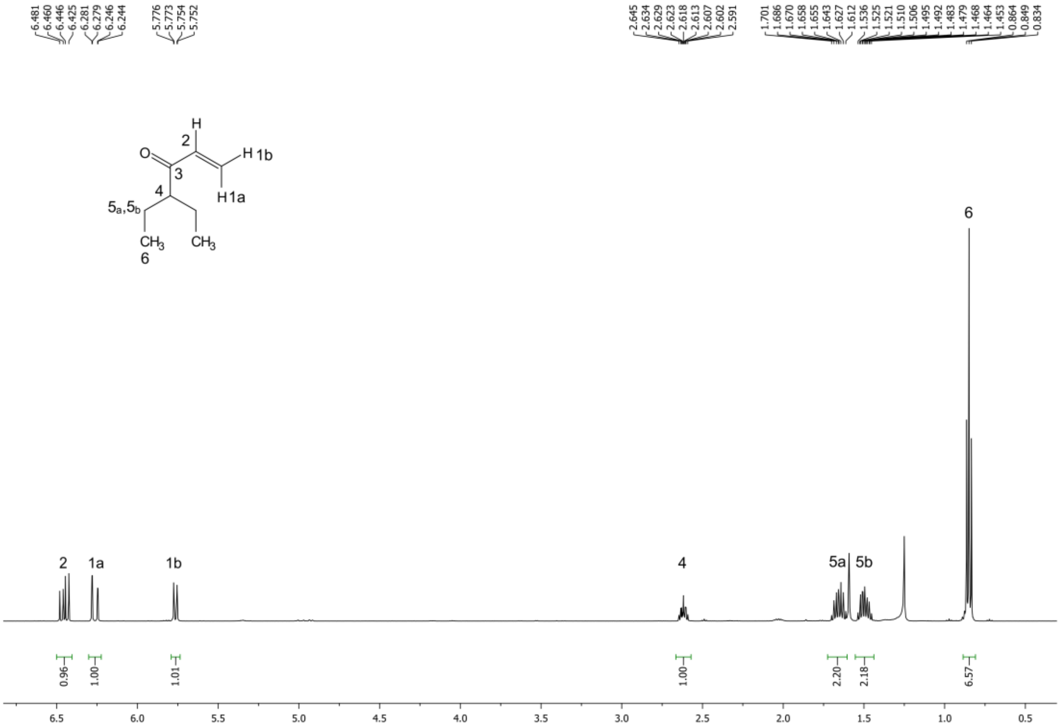

Figure S5. ^{1}H NMR (500 MHz, $CDCl_3$) spectrum of the *S. continentalis* extract together with chemical structure of julidone and assignations of its signals.

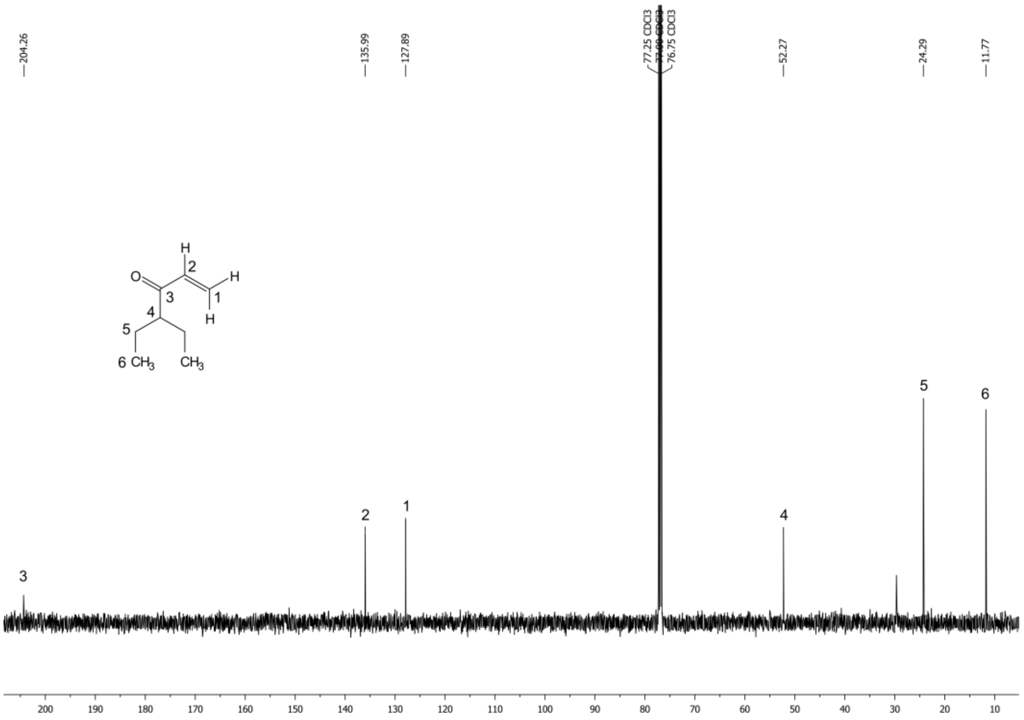

Figure S6. ^{13}C NMR (125 MHz, $CDCl_3$) spectrum of the *S. continentalis* extract together with chemical structure of julidone and assignations of its signals.

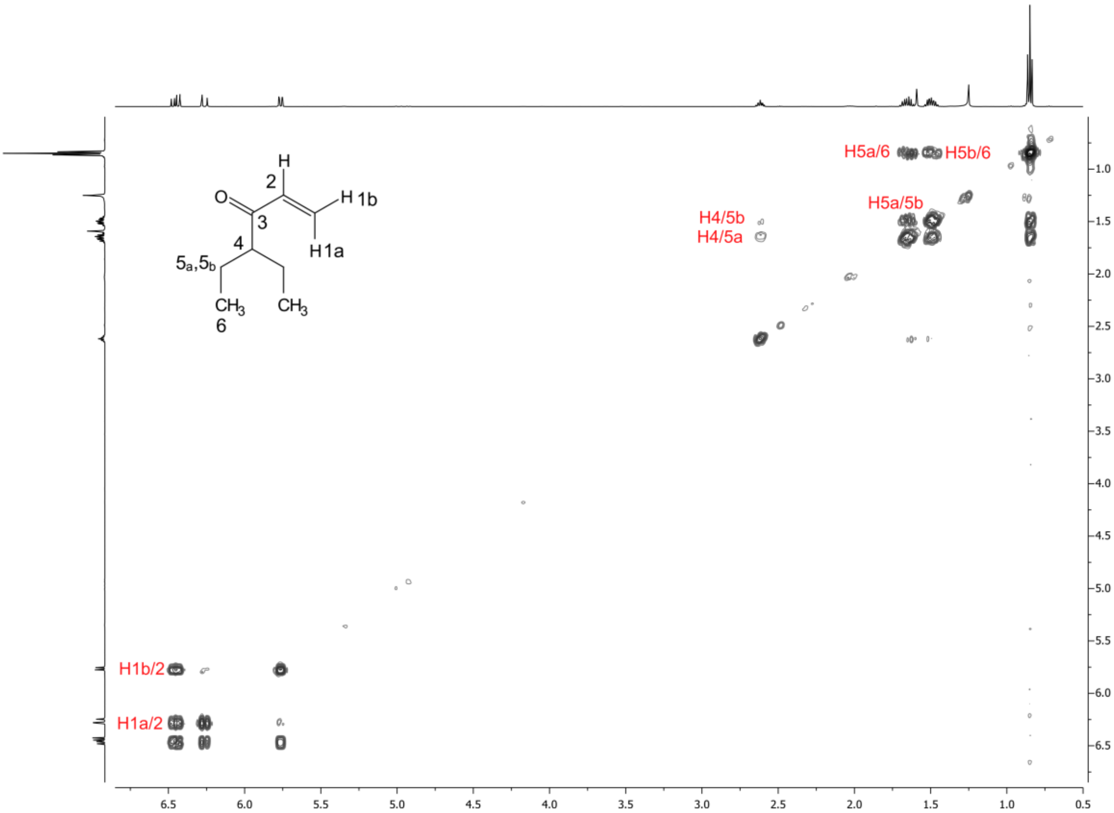

Figure S7. $^{1}H,^{1}H$-COSY (500 MHz, $CDCl_3$) spectrum of the *S. continentalis* extract, together with chemical structure of julidone and assignations of its signals.

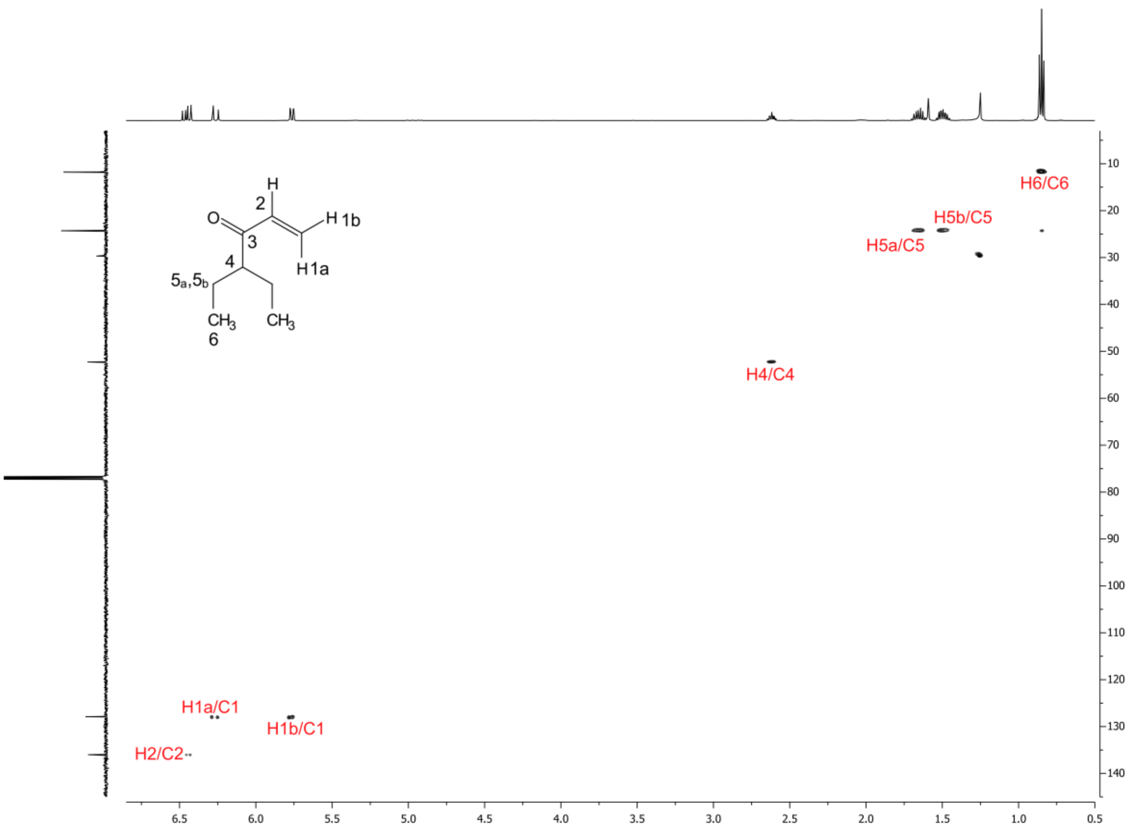

Figure S8. ^{1}H,^{13}C-HSQC (500 MHz, CDCl3) spectrum of the *S. continentalis* extract together with chemical structure of julidone and assignations of its signals.

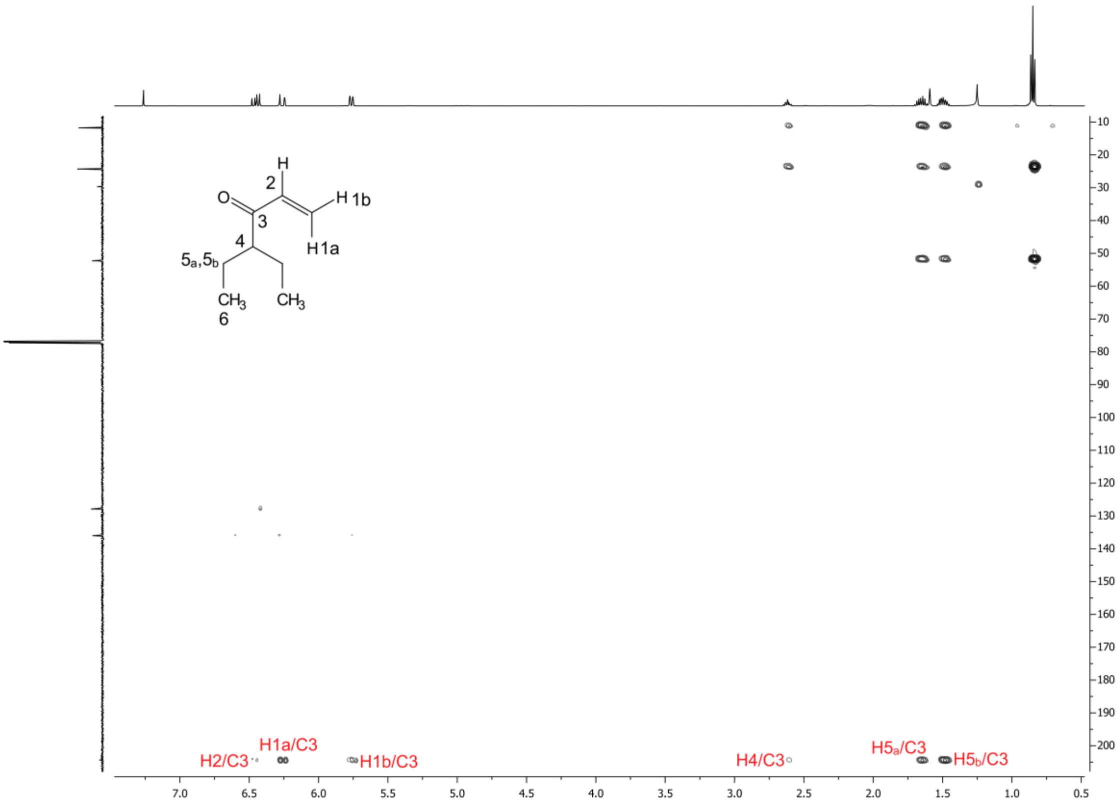

Figure S9. ^{1}H,^{13}C-HMBC (500 MHz, $CDCl_3$) spectrum of the *S. continentalis* extract, together with chemical structure of julidone and assignations of important correlations of C3 signal at 204.26 pmm.
